# Energy efficiency of quasi-monochromatic LEDs for road lighting under mesopic vision

**DOI:** 10.1038/s41598-026-53585-2

**Published:** 2026-06-08

**Authors:** Sławomir Zalewski, Krzysztof Skarżyński, Piotr Pracki

**Affiliations:** Lighting Technology Division, Electrical Power Engineering Institute, Warsaw University of Technology, Koszykowa 75, Warsaw, 00-662 Poland

**Keywords:** Light emitting diode (LED), Mesopic vision, Adaptation luminance, Road lighting, Energy efficiency, Energy savings, Engineering, Optics and photonics

## Abstract

The study addresses the energy efficiency of quasi-monochromatic LEDs for road lighting under mesopic vision conditions. It provides the outcome from the initial stage of research into the potential of such lighting at low adaptation levels. Outdoor electric lighting is widespread and essential for ensuring safety after dark, and current developments increasingly emphasize sustainable LED-based solutions. The existing standards define luminance levels for photopic vision, while nighttime conditions on illuminated roads fall within the mesopic range (0.005–5.000 cd/m^2^). The research aimed to determine potential of quasi-monochromatic LEDs and energy savings resulting from the use of the light sources in road lighting. Laboratory tests included LED chip samples with peak wavelengths between 495 and 605 nm. The analysis considered the spectral power distribution, S/P ratio and mesopic luminous efficacy of the LEDs, normalized power density and annual energy density for road lighting under mesopic vision conditions. The results confirmed that quasi-monochromatic LEDs are an alternative worth studying for mesopic vision conditions and can improve the energy efficiency of road lighting. For LEDs emitting within 495–570 nm range, mesopic luminous efficacy increased as adaptation luminance decreased. The analysis also showed that, compared with HPS lamps, a 570 nm LED and a mixed spectrum combining 2884 K and 500 nm LEDs may offer up to 20% higher energy efficiency at the lowest lighting levels (0.3–0.5 cd/m^2^). Consequently, annual energy savings in road lighting may reach approximately 20% relative to HPS lamps and even 30% relative to a typical white LED.

## Introduction

### Outdoor lighting issues and basic quantitative requirements

The use of electric lighting at night in outdoor areas is now ubiquitous. Night-time lighting enables people to function in outdoor environments^1^ as well as on roads and other traffic zones^2^. It is used to perform work tasks^3^ and to ensure safe movement^4^. Night-time illumination contributes to enhancing the urban environment^5^ and to increasing the sense of security^6^. It can also serve as a tool through which policymakers can influence community well-being^7^. In addition, the implementation of sustainable lighting solutions is of critical importance^8^. For this reason, current analyses of lighting solutions emphasize the combination of high-quality lighting conditions, energy efficiency, environmental protection, durability, and the high quality and reliability of lighting equipment^9–11^. Modern lighting systems predominantly employ electroluminescent light sources^12^. LED products have become globally widespread and widely accessible^13^, primarily because LED-based systems can consume significantly less energy than conventional lighting systems^14,15^. Sustainable lighting also involves the use of recyclable materials with reduced environmental impact^16^ and products manufactured using 3D printing technologies^17^. Moreover, diverse lighting control system topologies are increasingly being implemented^18^, and architects are more frequently accounting for the need to utilize daylight in public buildings efficiently^19^. Growing attention is also being paid to lighting systems tailored to user needs^20^ and to their circadian rhythms through the appropriate shaping of spectral distributions and circadian lighting parameters^21,22^. Sustainable lighting systems aim to both preserve the natural environment with minimal disturbance and improve user comfort and safety, thereby supporting human health. White light is typically used for general outdoor and road lighting. However, in the LED era, new concepts are emerging that use more monochromatic, highly saturated light, such as red^23^ or green^24^, to reduce environmental impact. Furthermore, there is also significant interest in amber LED lighting^25^.

Economic and environmental considerations limit the use of electric lighting outdoors at night. National legal regulations determine the extent of outdoor lighting. Lighting costs constitute a significant criterion in selecting a lighting solution. Economic analysis must consider investment costs, operating costs, and life-cycle costs^26–28^. Environmental aspects should also be considered, including the use of natural resources for energy production, greenhouse gas emissions, disposal of lighting equipment at the end of its life, and light pollution^29–31^.

Requirements for night-time lighting are regulated by national legal documents, including lighting standards as well as industry or informal documents such as LEED^32^, BREEAM^33^, and WELL^34^ certifications. In European Union countries, the formal requirements for the lighting of outdoor spaces are specified in standards EN 12464-2 (exterior lighting)^35^, EN 12,193 (sport lighting)^36^, and EN 13,201 (road lighting)^37^. These standards must also be considered in architectural or decorative lighting. However, due to its different nature, additional recommendations such as those of the International Commission on Illumination (CIE) are typically applied^38^. The requirements and recommendations regarding lighting conditions in areas used by vehicles and pedestrians indicate potential levels of average illuminance or luminance that occur at night (Table 1).

In most outdoor situations, the ability to distinguish fine details or colors at night is not a primary criterion. Therefore, meeting human needs for night-time illumination generally does not require providing either “high” illuminance or luminance levels, or high-quality color rendering of illuminated objects. Although significant focus on energy efficiency in outdoor lighting is evident, it must be emphasized that luminous efficacy and energy consumption are not the only critical factors in outdoor and road lighting. Equally important are the primary reasons for implementing lighting: ensuring safety and visual comfort, reducing glare, and enabling recognition of signs and faces^39^.

**Table 1:** Example ranges of maintained average illuminance and luminance levels based on standards^35,37^.

$\:{E}_{v,p}\left[lx\right]$	$\:{L}_{v,p}[cd/{m}^{2}]$
Illuminance on reference surface (P lighting class - footways, cycleways, pedestrian or cyclists streets, residential roads)	Luminance of road surface (M lighting class - motorized vehicles on traffic routes)
2–15	0.3–2
Illuminance on reference surface (pedestrian walkways-passages, parking areas)	Luminance of building facade
5–50	0–25
Illuminance on reference surface (C lighting class - motorized vehicles and other road users, on conflict areas such as shopping streets or road intersections)	Luminance of signs
7.5–50	50 − 1000

### Mesopic vision issue

The main issue with the lighting requirements mentioned in subsection 1.1 is that the light levels (luminance and illuminance) specified in standards refer to photopic vision conditions. However, at night, people perceive objects and their surroundings under different lighting conditions, including mesopic and scotopic, particularly in road lighting and outdoor areas, which are now illuminated mainly by LEDs.

Due to the adaptive ability of the human visual system, people can cope with both nighttime and daytime environments. In “dark conditions,” at luminance levels below 0.005 cd/m², vision is supported solely by rods. Under these conditions, the human visual system is in a state of scotopic vision^40^. In such lighting conditions, only general spatial orientation is possible, without the ability to discern details or colors^41^. In “bright conditions” (luminance of at least 5 cd/m²), vision is driven by cones. In this case, the human visual system is in a state of photopic vision^42^. Under such lighting conditions, fine details can be perceived, and colors can be distinguished. An intermediate state, in which rods and cones work simultaneously, is called mesopic vision^43^. It usually occurs at dusk, dawn, or in dimly lit areas (ambient luminance between 0.005 cd/m² and 5 cd/m²), such as roads or other outdoor workplaces (parking lots, urban parks, etc.). Rods are extremely sensitive to light and are responsible for vision in darkness, but they do not distinguish colors. Cones, on the other hand, are responsible for color vision and visual acuity but function effectively only at higher light levels. In mesopic vision, their functions partially complement each other, allowing limited color perception and moderate visual acuity. The luminous efficiency (spectral sensitivity) of the eye shifts toward shorter wavelength. It is the Purkinje effect, which stands that blue-green colors appear brighter than red^44^. This mechanism is crucial for drivers and pedestrians at low-light levels. Mesopic vision is difficult to assess accurately because it depends on the level of adaptation luminance and eye adaptation.

One of the fundamental parameters used to determine the suitability of a chosen light source for outdoor applications is the S/P ratio, described by Eq. (1). It is defined as the quotient of the scotopic luminous flux, represented by Eq. (2), and the photopic luminous flux, represented by Eq. (3).

1 $$\:S/P=\frac{{\phi\:}_{s}}{{\phi\:}_{p}}$$

2 $$\:{\phi\:}_{s}={K}_{S}\underset{380}{\overset{780}{\int\:}}{\phi\:}_{e}\left(\lambda\:\right)V'\left(\lambda\:\right)d\lambda\:$$

3 $$\:{\phi\:}_{p}={K}_{p}\underset{380}{\overset{780}{\int\:}}{\phi\:}_{e}\left(\lambda\:\right)V\left(\lambda\:\right)d\lambda\:$$

*where*:

$$\:S/P-scotopic\:to\:photopic\:index\:[-]$$

$$\:{\phi\:}_{s}-scotopic\:luminous\:flux\:\left[lm\right]$$

$$\:{\phi\:}_{p}-photopic\:luminous\:flux\:\left[lm\right]$$

$$\:{\phi\:}_{e}\left(\lambda\:\right)-spectral\:power\:distribution\:of\:radiant\:flux\:[W/nm]$$

$$\:{K}_{s}-scotopic\:radiation\:equivalent\:equal\:to\:1699\:lm/W$$

$$\:{K}_{p}-photopic\:radiation\:equivalent\:equal\:to\:683\:lm/W$$

$$\:{V}^{{\prime\:}}\left(\lambda\:\right)-scotopic\:luminous\:efficiency\:of\:the\:human\:eye\:[-]$$

$$\:V\left(\lambda\:\right)-photopic\:luminous\:efficiency\:of\:the\:human\:eye\:[-]$$

$$\:\lambda\:-wavelenght\:\left[nm\right]$$

**Fig. 1:**
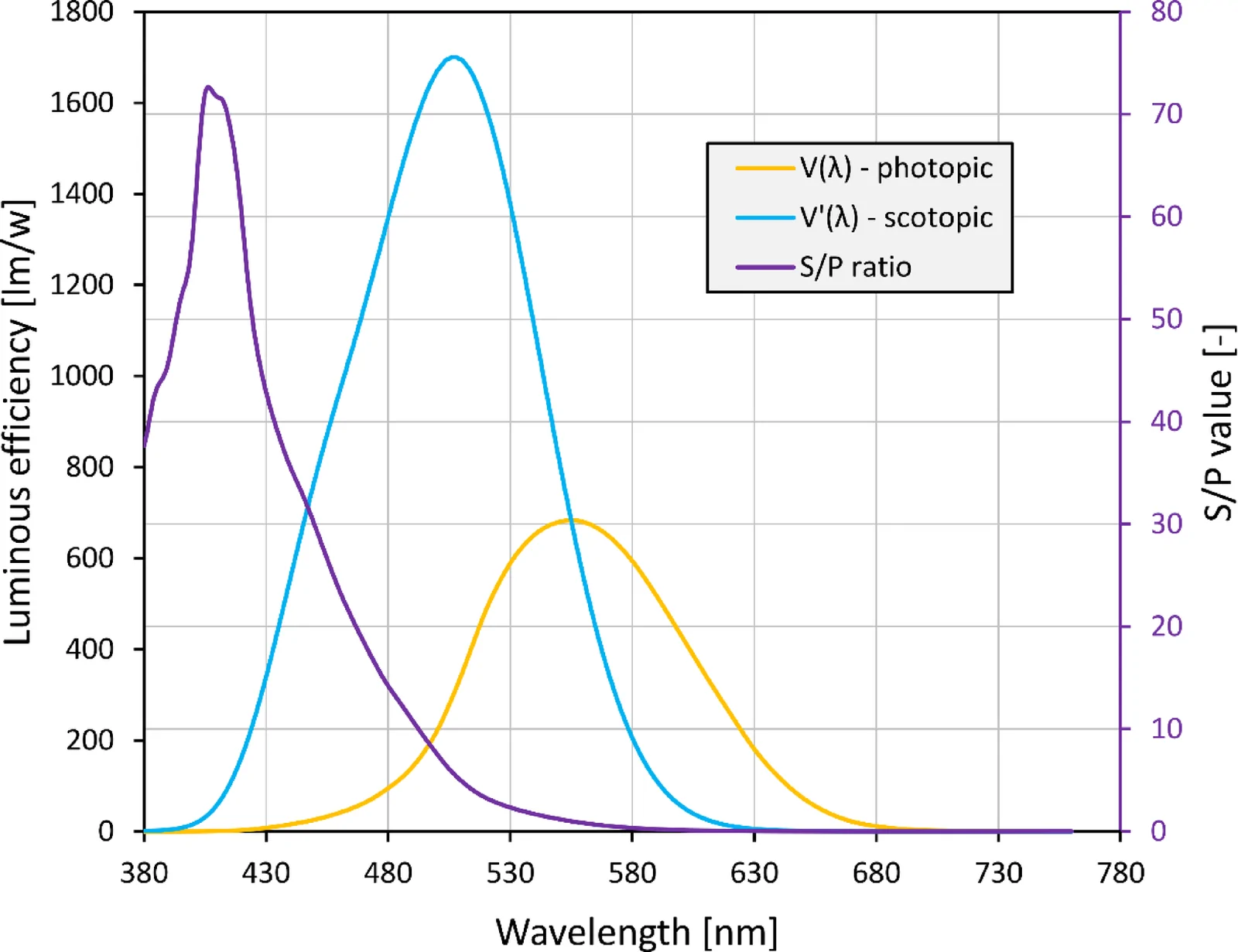
Characteristics of the absolute values (left scale) of the photopic and scotopic efficiency of the human eye and the S/P ratio (right scale) for monochromatic light as a function of wavelength.

The S/P ratio takes values from two semantically opposite ranges: from 0 to 1 or above 1. The lower the S/P ratio, the less suitable a given light source is for outdoor applications at illuminance levels corresponding to mesopic vision^45^. Values of this ratio greater than one indicate that the spectral distribution of the radiation creates the impression that, at exceptionally low lighting levels, the illumination appears brighter than would be expected based on its calculated photopic parameters.

Since our research focuses on assessing the potential for improving lighting energy efficiency in mesopic vision conditions by using quasi-monochromatic light, it is necessary to analyze how different colors are perceived by humans individually (when viewed separately). To achieve this, it is necessary to supplement knowledge of the photopic and scotopic luminous efficacy of individual wavelengths with information on the relationship between these two functions and to determine the S/P ratio function for monochromatic light as a function of wavelength. The integral functions for photopic and scotopic luminous flux reduce to a single, non-zero value for the current wavelength. The ratio (1) of the integral functions (2) and (3), being non-zero only for the current wavelength, then reduces to the quotient of two numbers. For each wavelength, the S/P value can be determined. The purple curve in Fig. 1 illustrates the variation of this feature as a function of wavelength. Therefore, the S/P ratio reaches highest value for monochromatic radiation at noticeably short wavelengths within the visible range (below 430 nm). However, this does not imply that such light is the most suitable for lighting under conditions of very low adaptation luminance. Radiation at these wavelengths has low luminous efficacy, meaning that a high power is required to perceive anything. The S/P ratio should not be analyzed in isolation, but only in conjunction with luminous efficacy.

The S/P ratio is only an indicative measure. It provides little information about the extent to which a spectral power distribution (SPD) of radiation influences the perception of brightness at low lighting levels. Complete information is provided only by the characteristics of mesopic luminous flux changes as a function of the adaptation level expressed by the photopic adaptation luminance. Mesopic luminance is expressed by Eq. (4) and it indicates how bright surfaces will appear under specific visual conditions when illuminated by a given spectral power distribution.

CIE Report 191^39^ assumes that the contributions of the photopic and scotopic vision to mesopic vision vary as a function of adaptation luminance within the range of 0.005 cd/m² to 5 cd/m² and is given by Eq. (5). It is expressed by the value of the adaptation coefficient m, which equals zero for an adaptation luminance of maximum 0.005 cd/m², and one for an adaptation luminance of minimum 5 cd/m². It means that the relative spectral sensitivity of the human visual system in mesopic vision represents a weighted average of the eye’s photopic and scotopic luminous efficiencies.

4 $$\:{L}_{v,m}=\frac{683}{{V}_{mes}({\lambda\:}_{0}=555nm)}\underset{380}{\overset{780}{\int\:}}{L}_{e}\left(\lambda\:\right){V}_{mes}\left(\lambda\:\right)\:d\lambda\:$$

5 $$\:{V}_{mes}\left(\lambda\:\right)=mV\left(\lambda\:\right)+\left(1-m\right)V{\prime\:}\left(\lambda\:\right)$$

where:

$$\:{L}_{v,m}-mesopic\:luminance\:[cd/{m}^{2}]$$

$$\:{L}_{e}\left(\lambda\:\right)-radiance\:\left[W{sr}^{-1}{m}^{-2}\right]$$

$$\:{V}_{mes}\left(\lambda\:\right)-mesopic\:luminous\:efficiecny\:of\:the\:human\:eye\:for\:the\:given\:adaptive\:luminance\:[-]\:$$

$$\:{V}^{{\prime\:}}\left(\lambda\:\right)-scotopic\:luminous\:efficiecny\:of\:the\:human\:eye\:[-]$$

$$\:V\left(\lambda\:\right)-photopic\:luminous\:efficiecny\:of\:the\:human\:eye\:[-]$$

$$\:m-adaptation\:coefficient\:[-]$$

$$\:\lambda\:-wavelenght\:\left[nm\right]$$

Road lighting is one of the most essential applications in which nighttime lighting conditions expose observers to luminance levels at mesopic vision. However, real road lighting situations are complex, and the luminance distribution within the driver’s visual field varies across space and in time^46^. Luminance levels typical of mesopic vision occur, but high luminances, typical of photopic vision, are also present. These originate from traffic signs, advertisements, shop windows, luminaires, or the headlights of oncoming vehicles as also, extremely low luminances from the night sky or from unlit areas distant from the road exist.

The driver’s visual field includes not only the road but also its surroundings and elements of the vehicle’s interior. On rural roads without lighting systems or illuminated objects, the highest luminance typically occurs in the central visual field, originating from the headlights of oncoming vehicles. In contrast, the lowest luminance appears in the peripheral field of view. On a road equipped with lighting or with illuminated objects in the surroundings, the highest luminance originates not only from oncoming headlights but also from luminaires, advertisements, and the illumination of buildings or other structures. This luminance occurs both in the central and peripheral visual field. In such conditions, low luminance from the night sky and dark objects also appear in the driver’s field of view. As a result, luminance distribution in the driver’s visual field varies substantially.

The central and peripheral vision are critical for road traffic safety. A driver must be able to detect objects both on-axis and off-axis. Research on reaction time in off-axis vision under mesopic lighting levels has shown that significantly shorter reaction times occur with SPD containing more short-wavelength radiation^47^. In this context, the S/P ratio is an effective parameter for studying how SPD influences reaction time.

Despite the considerable spatial and temporal variability of luminance levels and distributions in the nighttime visual field of drivers, road lighting luminances are essentially characteristic of mesopic vision^48^. Safety road traffic is primarily associated with ensuring drivers sufficient visual performance to effectively perceive objects on the road and in their surroundings under visual comfort^49^. The main factors influencing visual performance include object size and location in the visual field, object and background luminances (contrast), and the spectral characteristics of both the object and the background^50–52^. In addition to the geometric and spectral characteristics of the object and its background, the SPD of the light sources used in road lighting also affect the visual performance of drivers.

### The research aim and scope

Recent literature reports the effective use of light-emitting diodes (LED) with highly saturated red color in reducing light pollution^23,53^. Road lighting is also being thoroughly analyzed in terms of potential energy savings^54–56^. Taking into account the issue of mesopic vision, in which the maximum luminous efficacy shifts toward shorter wavelengths, a scientific problem arises concerning the effective use of quasi-monochromatic light in road lighting.

The primary objective of this research is to determine the potential levels of energy savings resulting from the application of road lighting that utilizes colored light (mainly quasi-monochromatic LED chips).

The scope of the work includes:

- laboratory studies of selected samples of quasi-monochromatic LED chips,
- calculations, and analysis of mesopic parameters and luminous efficacy,
- assessment of potential energy savings,
- identification of problems and limitations relevant to ongoing experimental research.

The work does not include the analysis of road contrast, glare, lighting uniformity, recognition of color elements in quasi-monochromatic lighting, and many other parameters that determine vision quality. It is due to the complexity of these studies, which require time and appropriate financial resources. However, such research is planned for the future, after first obtaining adequate information on the potential energy savings described in this study.

## Materials and methods

**Fig. 2:**
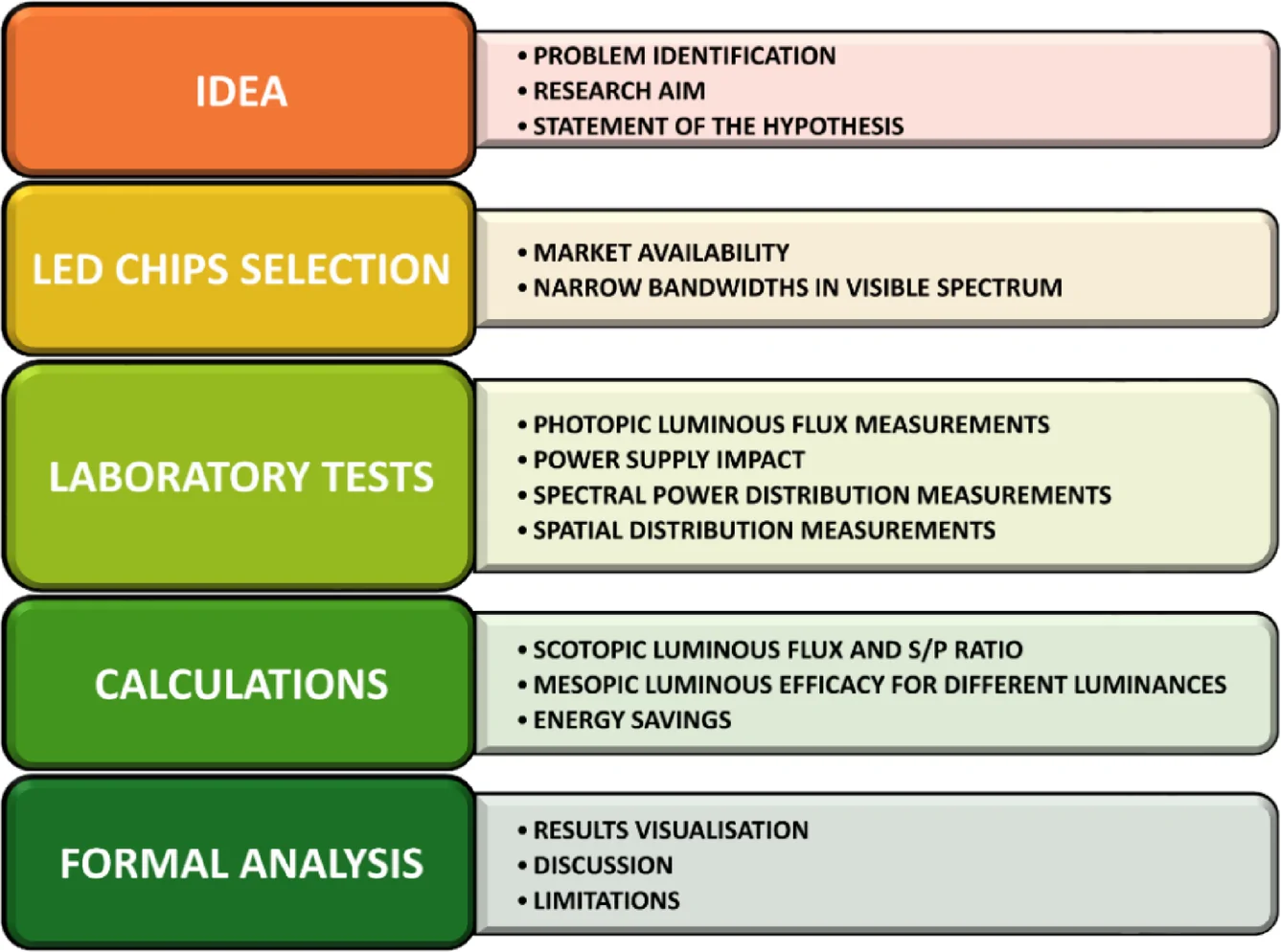
The procedure of the conducted research.

The procedure of the conducted research is presented in Fig. 2. First, the scientific problem was identified, and the aim and hypothesis of the study were defined. These were formulated and presented in Table 2.

**Table 2:** The problem, aim and hypothesis of conducted lighting research.

PROBLEM	Examining the influence of the spectral power distribution of quasi-monochromatic LED chips on the energy efficiency of road lighting, considering mesopic vision conditions.
AIM	Determining the level of electrical energy savings resulting from the use of quasi-monochromatic light in road lighting.
HYPOTHESIS	The use of LED chips with different, quasi-monochromatic spectral power distributions may improve the energy efficiency of road lighting.

In the next step, the availability of suitable LED chips was examined, and a set of nine samples was selected for testing. LED chips with different peak wavelengths covering almost entire visible spectrum were chosen. The selected set consisted of six types of LEDs, with five pieces of each type: 495 nm, 500 nm, 520 nm, 570 nm, 595 nm, and 605 nm. All LEDs except the 570 nm type are quasi-monochromatic sources, in which visible radiation is generated directly within the semiconductor junction of the crystal using a proper phosphor coating^57^. In contrast, the 570 nm LED has a structure similar to that of white LEDs (phosphor converted type). The core of the light source is a “royal blue” crystal emitting blue radiation with a dominant wavelength of 435 nm. It is coated with a phosphor that converts this radiation into “lime-white” light with a peak wavelength of 570 nm. The rated power of all LEDs was approximately 1 W (current control fixed at 350 mA; the forward voltages across the junctions result from their internal resistance and range from 2.44 to 3.64 V). A summary of selected data characterizing the quasi-monochromatic LEDs used in the study is presented in Table 3.

**Table 3:** The selected technical spectral datasheet of light sources used for research (CIE 2-degree photometric observer, nominal operating conditions 350 mA after 30 min of short-term stabilization).

Light source	x [-]	x [-]	CCT [K]	Duv [-]	FWHM [nm]
LED 605 nm	0.0696	0.4381	-	0.001074	~ 20
LED 595 nm	0.0673	0.5295	-	0.004879	~ 19
LED 570 nm	0.4178	0.5196	-	0.045950	~ 114
LED 520 nm	0.1496	0.7135	-	0.163100	~ 34
LED 500 nm	0.5788	0.4181	-	0.172800	~ 27
LED 495 nm	0.6307	0.3678	-	0.162600	~ 26
LED 2884 K	0.4483	0.4075	2884	-0.000100	~ 130
LED 5049 K	0.3448	0.3580	5049	0.003300	~ 140
HPS 1908 K	0.5380	0.4128	1908	0.001000	~ 90

**Fig. 3:**
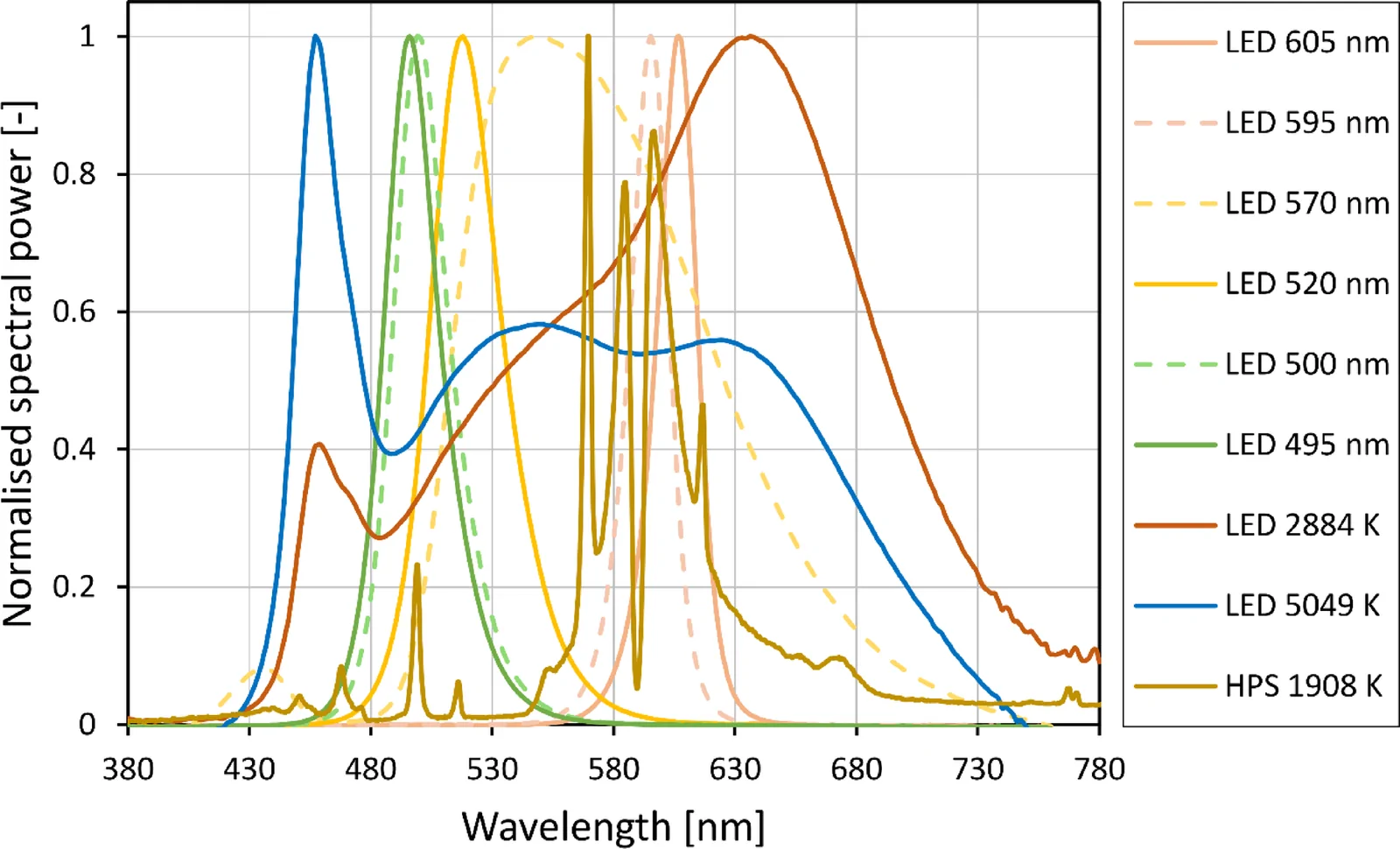
Normalized spectral power distributions of the selected LED chip samples.

Additionally, for comparison purposes, two white LEDs with correlated color temperatures (CCT) of 2884 K and 5049 K, and high color-rendering indices above 80, were selected, as well as a typical high-pressure sodium (HPS) lamp with CCT about 1908 K and CRI of 22. The normalized SPDs of the selected, nine LED chip types are presented in Fig. 3.

**Fig. 4:**
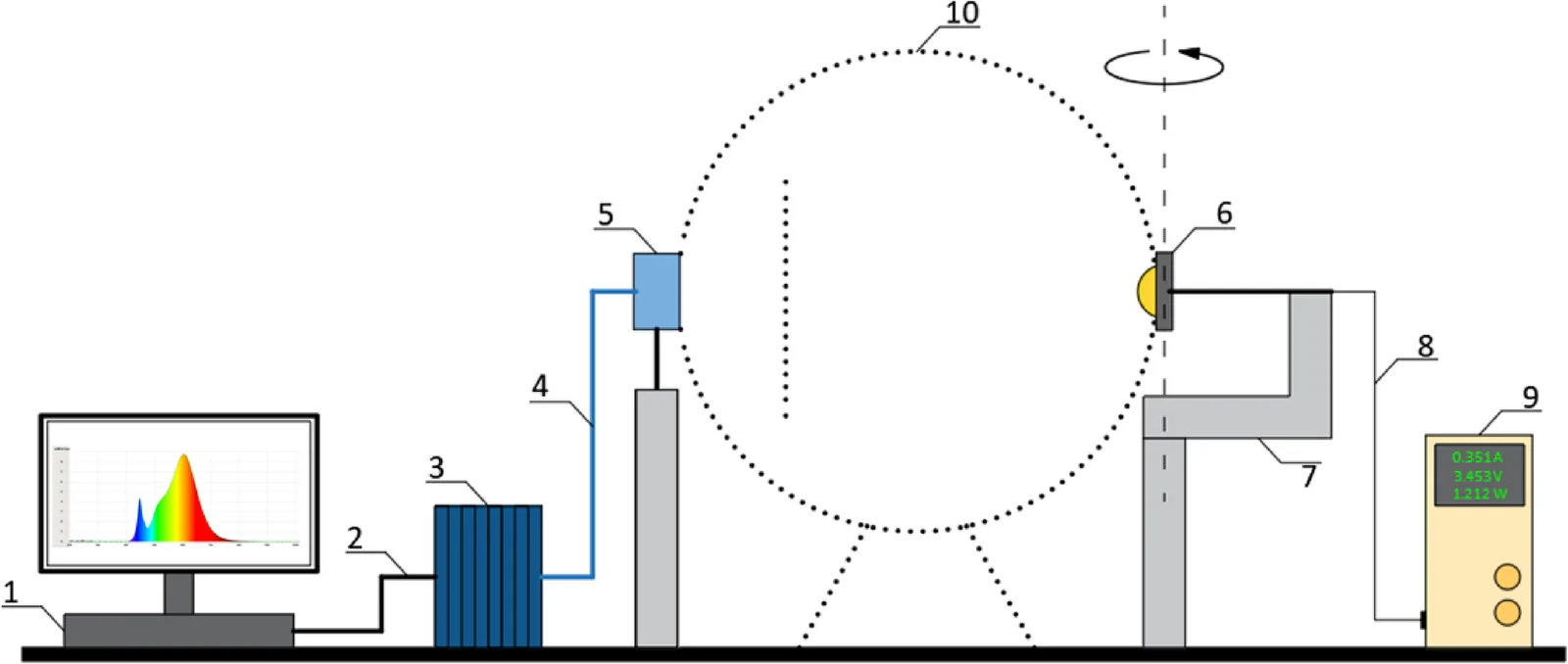
Schematic of the laboratory measurement setup: 1 – computer with software, 2 – communication wire, 3 – spectroradiometer, 4 – optical fiber, 5 – measurement head, 6 – test sample, 7 – goniometer, 8 – power wires, 9 – programmable DC power supply, 10 – integrating sphere.

After selecting the set of samples, laboratory tests were carried out in a photometric laboratory. The measurement setup presented schematically in Fig. 4 was used in a darkroom. All measurements of the SPDs were performed using a GL Optic Spectis 4.0 spectroradiometer (https://gloptic.com/products/gl-spectis-4-0-m/; *Retrieved on 24 November 2025*). The data were processed and collected using the manufacturer’s dedicated software. The study also utilized a calibrated integrating sphere and a programmable RIGOL DP 832 A DC power supply (https://rigol.com.pl/pl/p/Programowalny-Zasilacz-Rigol-DP832A/35; *Retrieved on 24 February 2026*), ensuring high stability of the power supplied to each sample, and consequently, the stability of the measured photometric and colorimetric parameters. At this point, all issues related to the thermal stability of the tested LED sources were resolved. The individual chips were mounted on appropriate heat sinks and underwent short-term stabilization. This process lasted 20–30 min, and the final measurements were taken only when the signal level (luminous flux) did not vary by more than a few lumens across three consecutive readings.

Each sample was evaluated at various currents, ranging from 50 mA to 350 mA, with the step od 50 mA. It was done to verify the repeatability of the results and ensure the validity of subsequent analysis and calculations of the relevant photometric parameters. Additionally, luminous intensity distribution (LID) curves of the quasi-monochromatic LED chips were measured. It was achieved using a miniature goniometer, after removing the LED from the integrating sphere setup. The goniometer allowed angular adjustment of the LED chip relative to the spectroradiometer sensor in 5° increments, with a measurement distance of 50 cm.

The LID curves were plotted for each quasi-monochromatic LED chip. This procedure was conducted to provide a comprehensive characterization of the quasi-monochromatic LED chips, enabling analysis of their potential use in the optical systems of road lighting luminaires.

The primary output obtained from the laboratory tests consisted of the SPDs of the tested LED chips, measured with a 1 nm step. These data were used to determine parameters such as luminous flux and luminous efficacy for both photopic and mesopic vision. In the case of mesopic vision, the luminous flux value varies with the adaptation luminance level. Therefore, its values were calculated for different adaptation levels. A logarithmic progression of adaptation luminance values was adopted. Calculations were performed for adaptation luminance: 0.005, 0.01, 0.02, 0.05, 0.1, 0.2, 0.5, 1.0, 2.0, and 5.0 cd/m² of photopic luminance.

From the SPD of a given light source, the photopic luminous flux, scotopic luminous flux, and the S/P ratio were calculated. Photopic and scotopic luminances were determined using Eqs. (6) and (7). Mesopic luminance for each level of photopic luminance was determined using the iterative method described in CIE Report 191^46^, described by the Eq. (8). While the mesopic luminous efficacy values were then calculated from the photopic luminous efficacy using Eq. (9). Equation 9 may not appear evident at first glance. However, it should be noted that it arises from the fact that both luminance and luminous efficacy are photometric quantities that are directly proportional to the luminous flux.

6 $$\:{L}_{v,p}={L}_{A}$$

7 $$\:{L}_{v,s}=\frac{{\phi\:}_{S}}{{\phi\:}_{P}}{L}_{v,p}$$

8 $$\:\left\{\begin{array}{c}{L}_{v,p}=\frac{{m}_{n-1}{L}_{v,p}+(1-{m}_{n-1}){L}_{v,s}V{\prime\:}\left(\lambda\:\right)}{{m}_{n-1}+(1-{m}_{n-1})V{\prime\:}\left(\lambda\:\right)}\\\:{m}_{0}=0.5\\\:{m}_{n}=0.767+0.334log\left({L}_{mes}\right)\end{array}\right.$$

9 $$\:{\eta\:}_{mes}=\frac{{L}_{v,p}}{{L}_{v,p}}\eta\:$$

where:

$$\:{L}_{v,p}-photopic\:luminance\:[cd/{m}^{2}]$$

$$\:{L}_{A}-adaptation\:luminance\:[cd/{m}^{2}]$$

$$\:{L}_{v,s}-\:scotopic\:luminance\:[cd/{m}^{2}]$$

$$\:{L}_{v,m}-\:mesopic\:luminance\:[cd/{m}^{2}]$$

$$\:{\phi\:}_{p}-photopic\:luminous\:flux\:\left[lm\right]$$

$$\:{\phi\:}_{s}-scotopic\:luminous\:flux\:\left[lm\right]$$

$$\:V\left(\lambda\:\right)-photopic\:luminous\:efficiecny\:of\:the\:human\:eye\:[-]$$

$$\:{V}^{{\prime\:}}\left(\lambda\:\right)-scotopic\:luminous\:efficiecny\:of\:the\:human\:eye\:[-]$$

$$\:{m}_{0}-initial\:adaptation\:coefficient\:from\:CIE191\:report\:equal\:to\:0.5$$

$$\:{m}_{n-1}-adaptation\:coefficient\:in\:step\:n-1\:$$

$$\:{m}_{n}-adaptation\:coefficient\:in\:step\:n$$

$$\:{\eta\:}_{mes}-mesopic\:luminous\:efficacy\:[lm/W]$$

$$\:\eta\:-photopic\:luminous\:efficacy\:[lm/W]$$

The corresponding parameter values obtained from the measurements and calculations are presented in the graphs and described accordingly. A discussion of the results was conducted, enabling an assessment of the potential for achieving high energy efficiency in quasi-monochromatic road lighting under various mesopic vision conditions. The potential energy savings resulting from the application of such solutions were also analyzed. All results presented in this article are supported by lighting calculations derived from the theory and practice of illuminating engineering. It applies particularly to the photometric parameters and energy efficiency, regarding their potential and dynamics^58^. The use of lighting simulation software, such as DIALux, was intentionally omitted. Outdoor lighting is highly complex, and numerous factors influence its energy efficiency. The most critical among these include the luminous intensity distribution (photometric web), the arrangement and aiming of luminaires, and the pole mounting height. The variety of these parameters and influencing factors could obscure potential energy savings resulting solely from the analyzed spectral issues and mesopic vision, thereby compromising the accuracy of the analysis and conclusions. In the end, the limitations of the study were described as well as the eventual implementation problems. The possible directions for further research and the entire research balance were also provided in a very concise way.

## Results and discussion

### Analysis of the performed laboratory measurements

For six different quasi-monochromatic LED chips (495 nm, 500 nm, 520 nm, 570 nm, 595 nm, and 605 nm), with five of each type, their SPDs, LIDs, active powers, and luminous fluxes were measured. The results were averaged for each LED type.

**Fig. 5:**
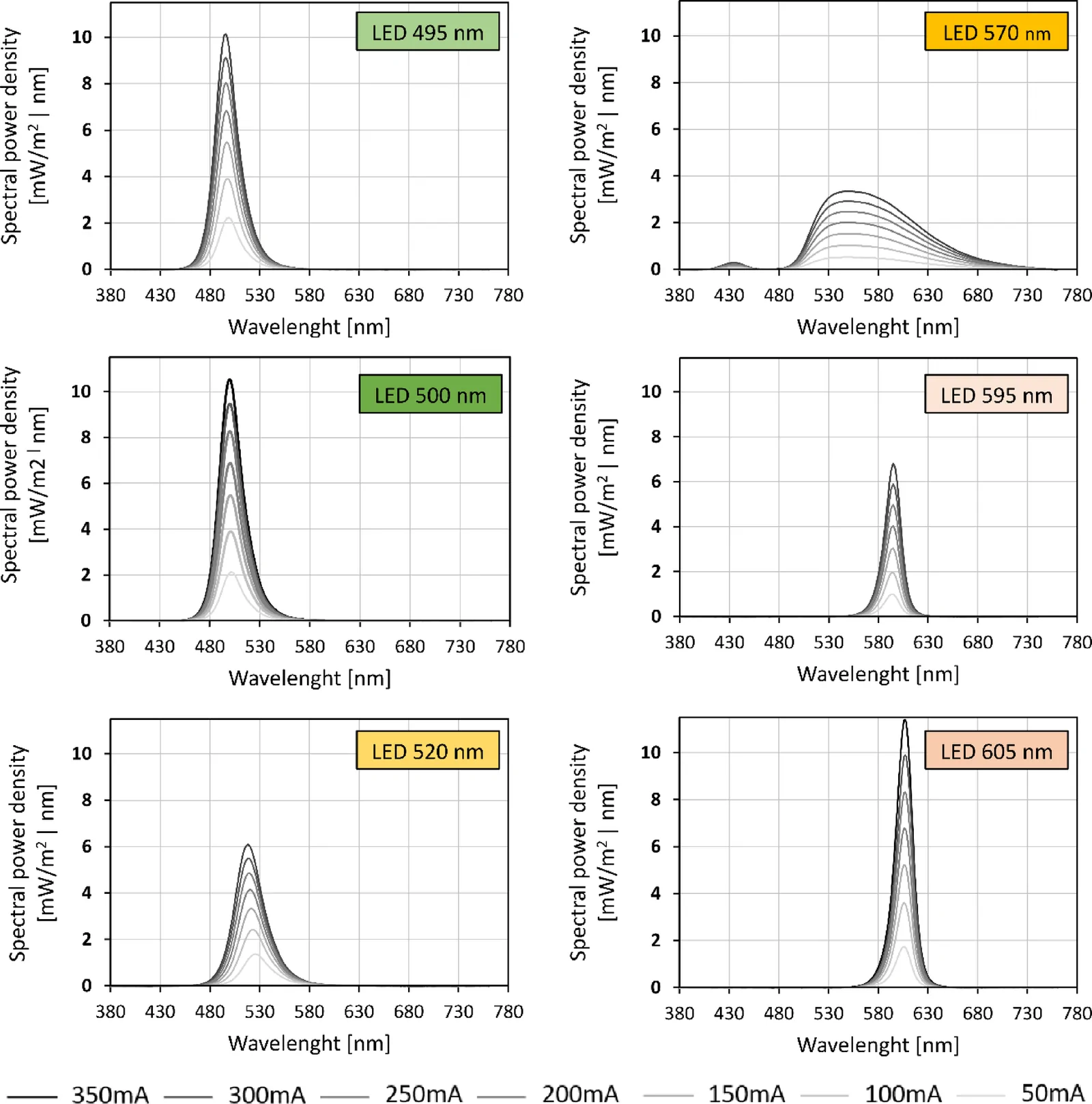
Spectral power density distributions of the quasi-monochromatic LEDs’ for different driving currents.

The averaged SPDs emitted by each type of LED as a function of current are shown in Fig. 5. The obtained spectrums indicate that each tested LED emits light within the intended range. The individual emission peaks correspond to the nominal wavelengths of 495 nm, 500 nm, 520 nm, 570 nm, 595 nm, and 605 nm, respectively. Increasing the current in the range of 50–350 mA enhances the maximum spectral power density and produces predictable changes in light intensity.

In almost all cases, the spectrum is relatively symmetric around the emission peak wavelength, regardless of the current value. No significant shifts in peak wavelength were observed in the SPDs for any LED chip except 520 nm. In this case, the maximum peak value is shifted by about 14 nm toward longer wavelengths for the lowest current (50 mA). It is insignificant when using high-quality constant-current power supplies. Generally, it is desirable that increasing the current affects only the intensity, not the spectral characteristics in most cases. Therefore, it does not alter the perceived color. So, it can be concluded that the tested LEDs exhibit good repeatability and predictable spectral behavior.

**Fig. 6:**
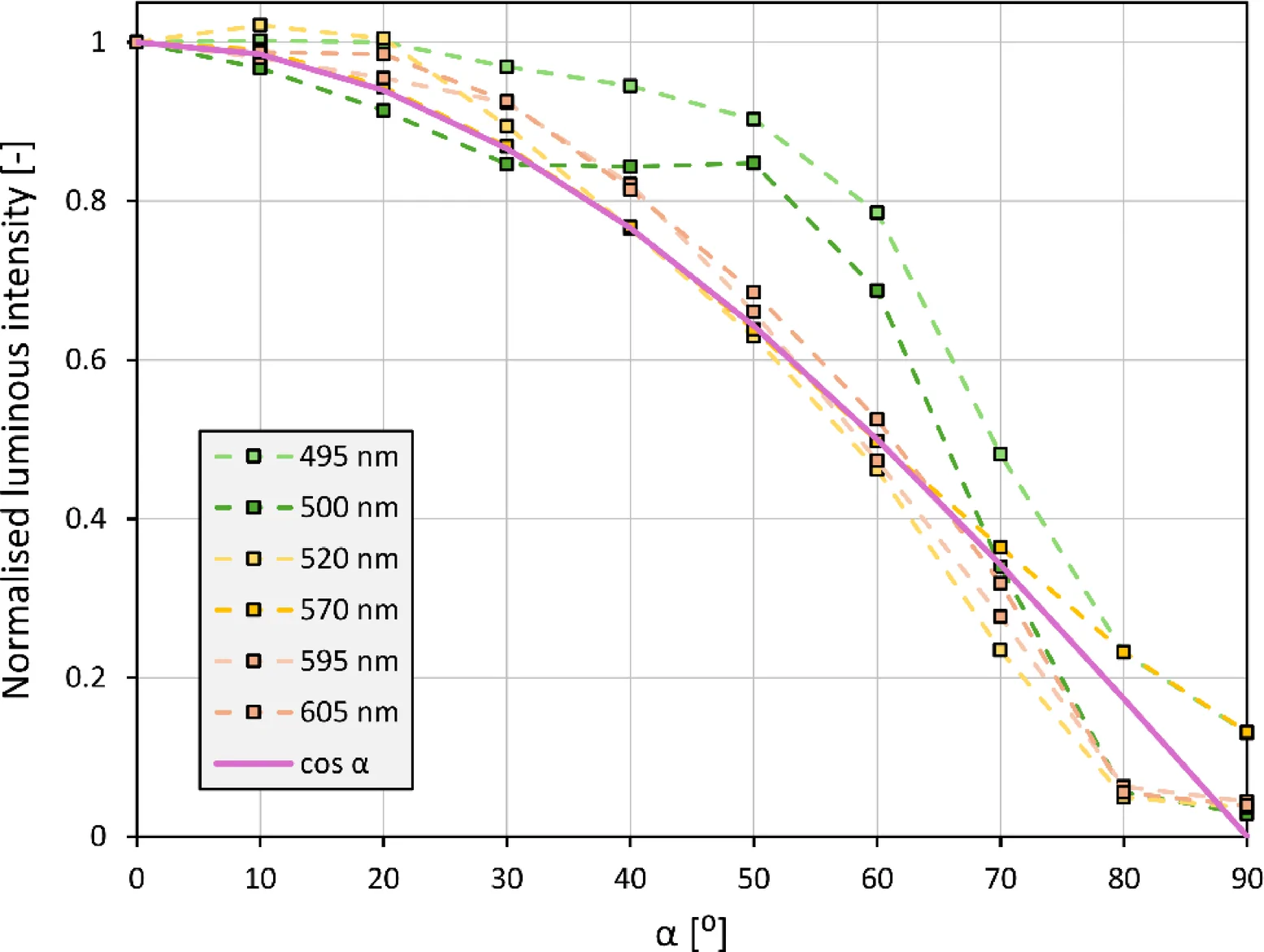
The luminous intensity distribution curves of selected color LED chips.

The LIDs at the maximum driving current (350 mA) are shown in Fig. 6. The radiation patterns of the individual tested quasi-monochromatic LEDs were compared with the theoretical Lambertian, cosine distribution. The fit to the cosine curve was assessed by comparing the areas under the curves using the trapezoidal rule for integration, and then relating the difference between these areas to the percentage of the area under the ideal cosine curve. The 605 nm and 595 nm LEDs exhibited very similar spatial distributions, consistent with Lambertian emission, with a calculated error of approximately 2%. For the 570 nm, 520 nm, and 500 nm LEDs, the error ranges from 5.5% to 8.5%. The poorest fit to the Lambertian distribution was obtained for the 495 nm LED, with approximately 15% deviation. The observed differences are primarily attributed to the type of chemical mixture used to generate visible radiation. However, these differences are not significant enough to prevent the production of similar photometric distributions for road lighting using the chosen LEDs. It is mainly due to the current advanced capabilities in designing complex optical systems for luminaires by simulation methods^59^.

**Fig. 7:**
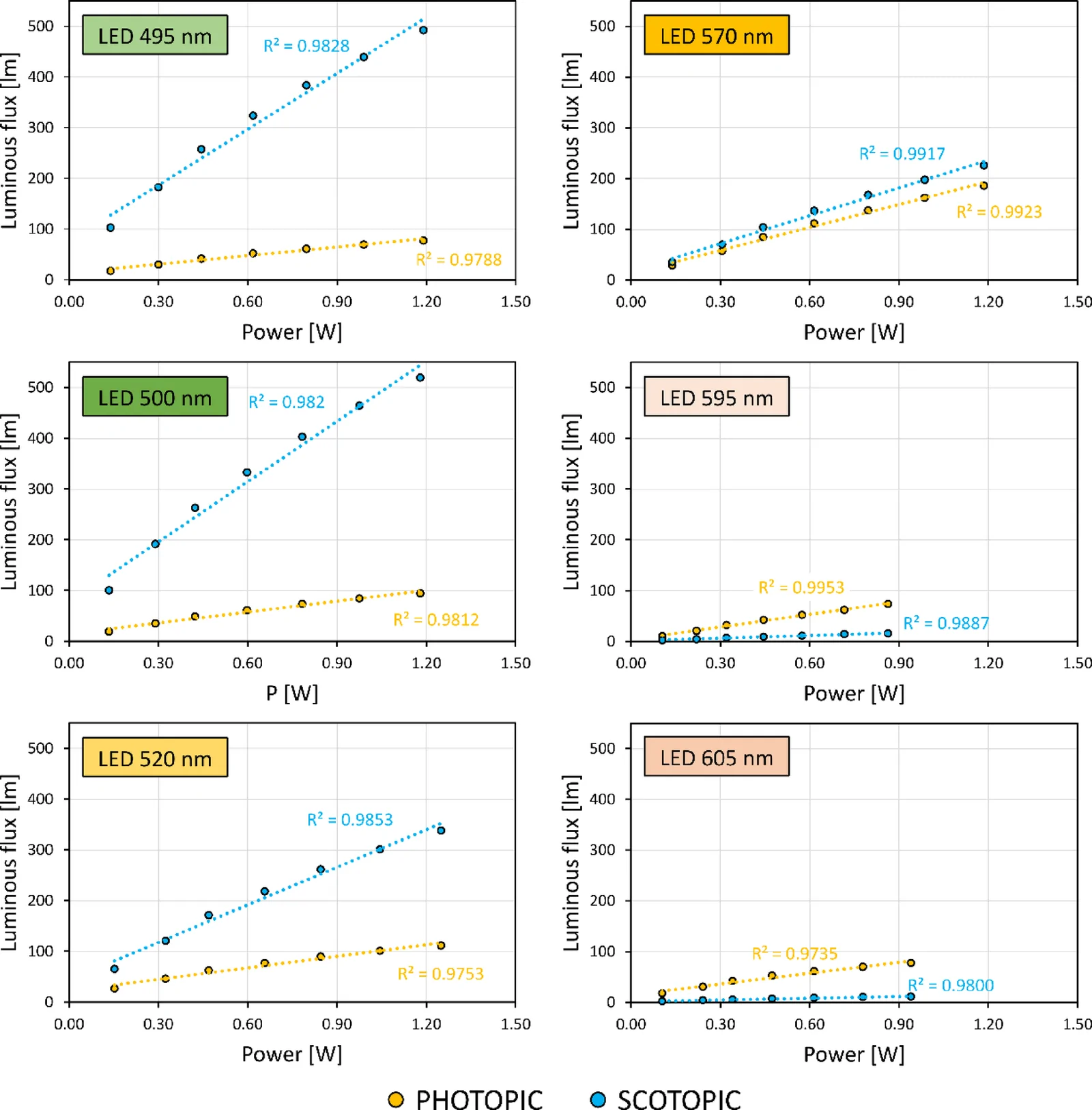
The luminous fluxes of selected color LED chips as a function of power.

The characteristics of the photopic and scotopic luminous flux variations as a function of active power for the different LED types are shown in Fig. 7. It should be noted that very strong linear correlations were obtained between luminous flux and active power in all cases. For the 495 nm, 500 nm, 520 nm, and 570 nm LEDs, the scotopic luminous flux exceeds the photopic luminous flux for all power levels from approximately 0.2 W to over 1.2 W. At 1.2 W, the highest scotopic luminous flux, exceeding 500 lm, was obtained for the 495 nm LED, while the lowest, around 200 lm, was for the 570 nm LED. The opposite trend is observed for the 595 nm and 605 nm LEDs. For these LEDs, the photopic luminous flux is always higher than the scotopic luminous flux, regardless of the electrical power supplied. These observations indicate a significant variability in the ratios of luminous fluxes depending on the SPD, the peak wavelength, and the current conditions. This behavior is consistent with theoretical predictions.

**Fig. 8:**
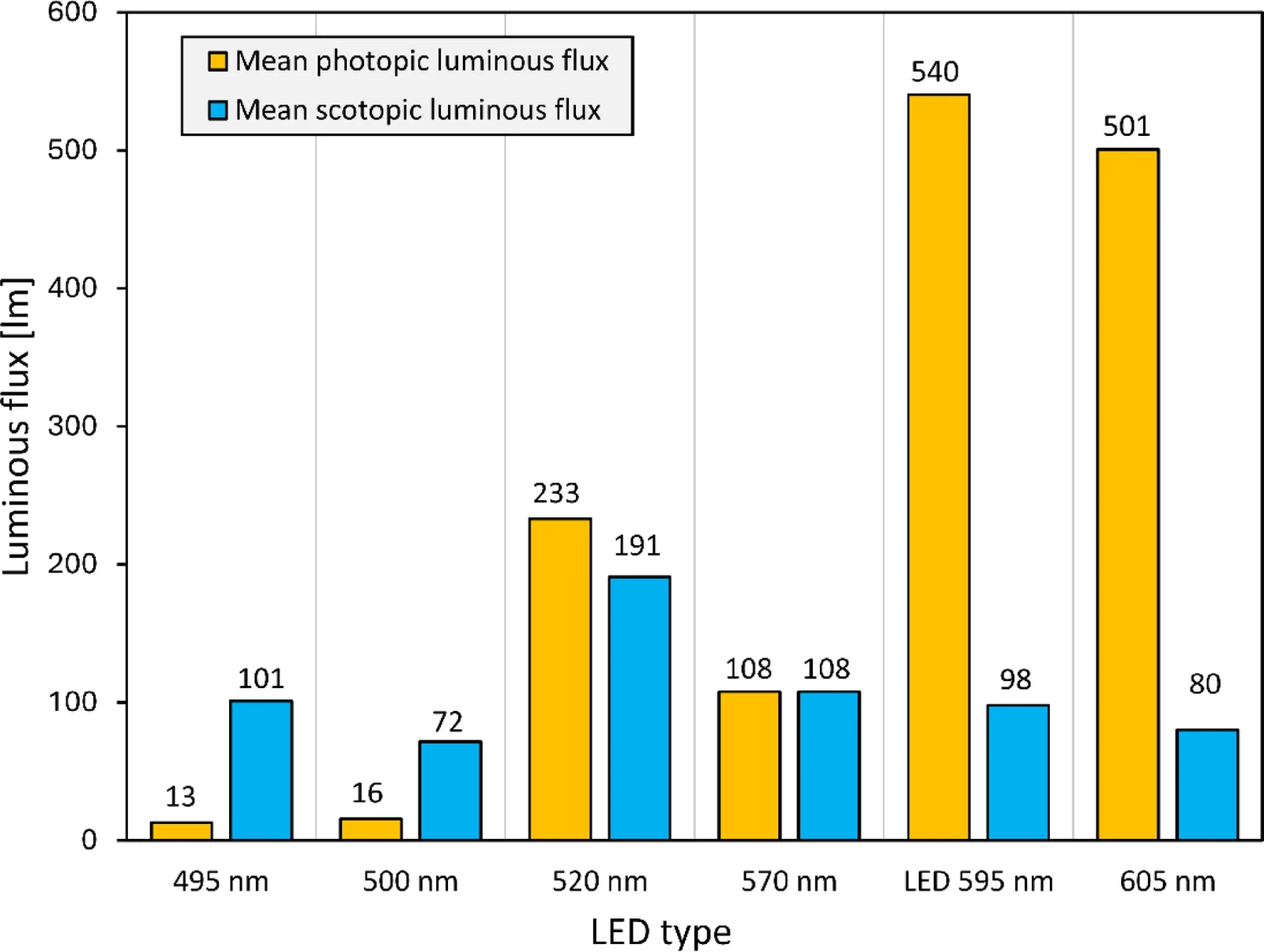
The average values of the luminous fluxes of selected quasi-monochromatic LED chips.

The relationships between photopic and scotopic luminous fluxes for the individual tested LEDs are even more clearly visible in Fig. 8. For LEDs with emission peaks at shorter wavelengths (495 nm and 500 nm), the scotopic luminous flux distinctly dominates. It is 4.5 to 7.5 times greater than the photopic luminous flux for these LEDs. It is directly due to the higher spectral sensitivity of the dark-adapted human eye.

Conversely, as the wavelength increases, the contribution of the scotopic flux decreases. For the 595 nm and 605 nm LEDs, the photopic luminous flux is on average about five times higher than the scotopic flux. The 570 nm LED represents a transitional point. The photopic and scotopic luminous flux values are equal. The obtained data preliminarily supports the proposed hypothesis: several types of quasi-monochromatic LEDs are, in terms of energy efficiency, better suited to prevailing lighting conditions in the luminous environment. However, it should also be evaluated deeper in terms of conventional energy efficiency approach and assessment methods.

### Analysis of the calculation results of the S/P index and radiation chromaticity

For each type of quasi-monochromatic LED chips, the S/P ratio was also determined using Eqs. (1), (2), and (3). The obtained S/P values are presented in Table 4. For comparison with conventional lighting solutions, spectrometric data from two white LEDs were used: a warm white LED with a CCT of approximately 2884 K and a cool white LED with a CCT of approximately 5049 K. Additionally, a high-pressure sodium (HPS) lamp with a CCT of around 1908 K was included. For these light sources, the S/P value was also determined and is presented in Table 4.

**Table 4:** The values of the S/P ratio *for selected light sources*.

LIGHT SOURCE	LED 495 nm	LED 500 nm	LED 520 nm	LED 570 nm	LED 595 nm	LED 600 nm	LED 2884 K	LED 5049 K	HPS 1908 K
S/P	6.26	5.51	3.06	1.22	0.22	0.13	1.42	2.15	0.56

The lowest S/P ratio value is 0.13, obtained for the 600 nm LED, while the highest S/P ratio is 6.26, for the 495 nm LED. Therefore, SPD of the 495 nm LED is more suitable for outdoor lighting, as high values of this ratio may contribute to a greater sense of safety for users^60^. Moreover, some studies on conventional light sources have suggested a potential reduction of up to 20% in energy consumption when using applications with a high S/P ratio^61^. The use of HPS lamps with an S/P ratio below 0.6 results in a slight degradation of the actual quality of vision at low ambient luminance levels compared to the calculated values. White LEDs (2884 K and 5049 K) are characterized by S/P ratios from about 1.42 to 2.15 respectively. It indicates that the 2884 K LED generates a perceived brightness that differs noticeably from the calculated values while the 5049 K LED produces a perceived brightness that is significantly higher than the calculated value.

**Fig. 9:**
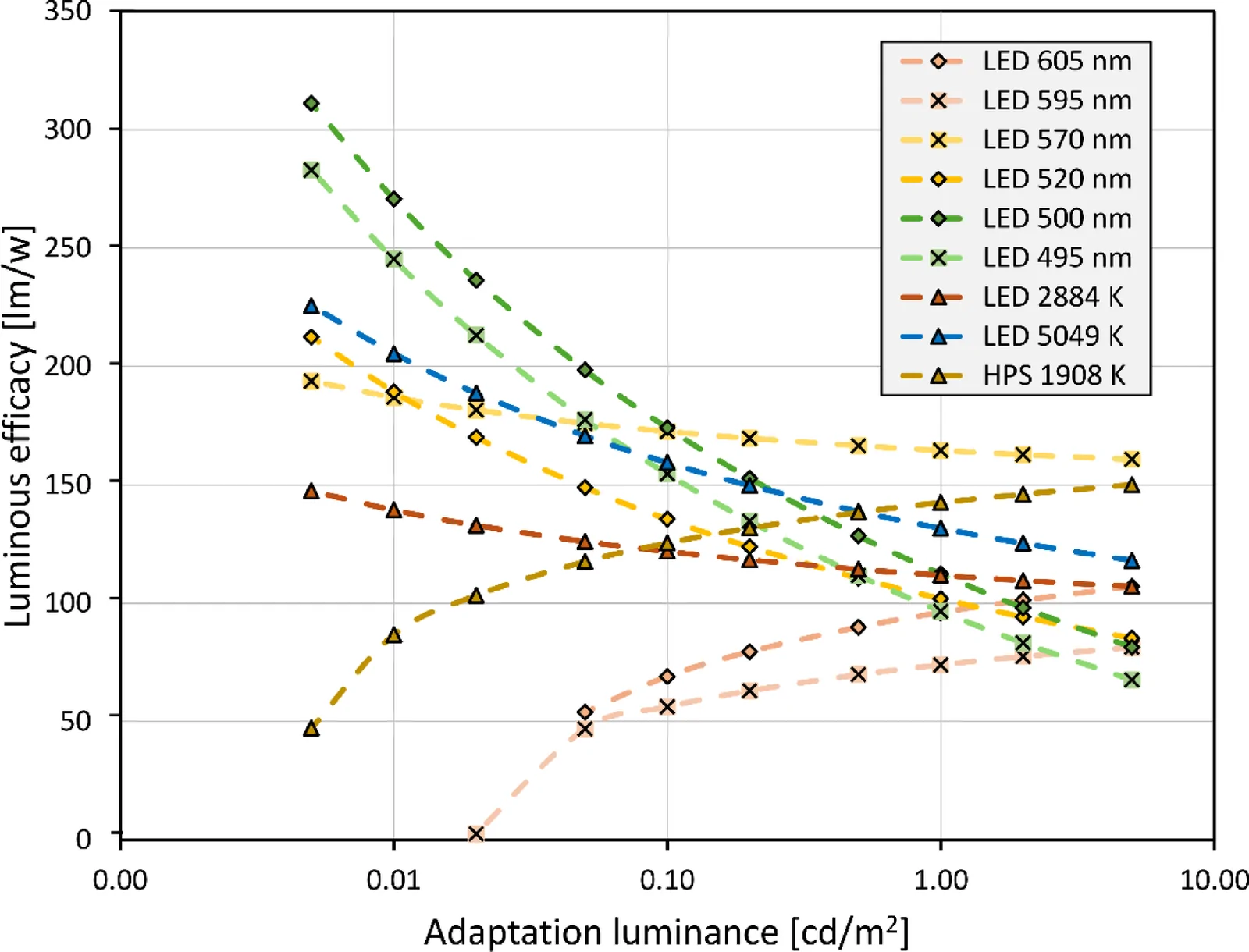
Mesopic luminous efficacies of the testes quasi-monochromatic LED chips as a function of adaptation luminance.

For the tested quasi-monochromatic LEDs, white LEDs, and the reference HPS lamp, mesopic luminances of the observer’s field of view were calculated as a function of photopic adaptation luminance using Eqs. (6), (7), (8), and (9). Subsequently, mesopic luminous efficacies were determined using Eq. (9). Thus, for each tested application, a characteristic of luminous efficacy as a function of adaptation luminance was obtained. These results are presented in Fig. 9.

As expected from the spectral sensitivity curves of the human eye adapted to light and dark conditions, it can be observed that the mesopic luminous efficacy of quasi-monochromatic LEDs with peak wavelengths above 555 nm decreases with decreasing adaptation luminance (Fig. 9). For LEDs with peak wavelengths of 570 nm and lower, an increase in mesopic luminous efficacy is observed as the adaptation luminance decreases. An interesting case is the LED chip labeled “570 nm” with lime-white color of emitted light. Despite a high photopic luminous efficacy of 160 lm/W, its mesopic luminous efficacy changes only slightly across a wide range of adaptation levels. A similar behavior is observed for the 2884 K LED, which emits heterochromatic warm light.

Under outdoor lighting conditions, particularly road lighting, adaptation levels are expected to be relatively high, falling within the upper part of the mesopic vision range. In the range above 0.1 cd/m² adaptation luminance, the high photopic efficacy has a greater influence on the resulting mesopic efficacy of most tested LEDs than the increase associated with high S/P values. A noticeable change in luminous efficacy is observed with varying adaptation levels in this range for LEDs with emission peaks at 495 nm, 500 nm, and 520 nm. It suggests that these LEDs may perform better in outdoor work area lighting in terms of achieving solutions characterized by high energy efficiency.

**Fig. 10:**
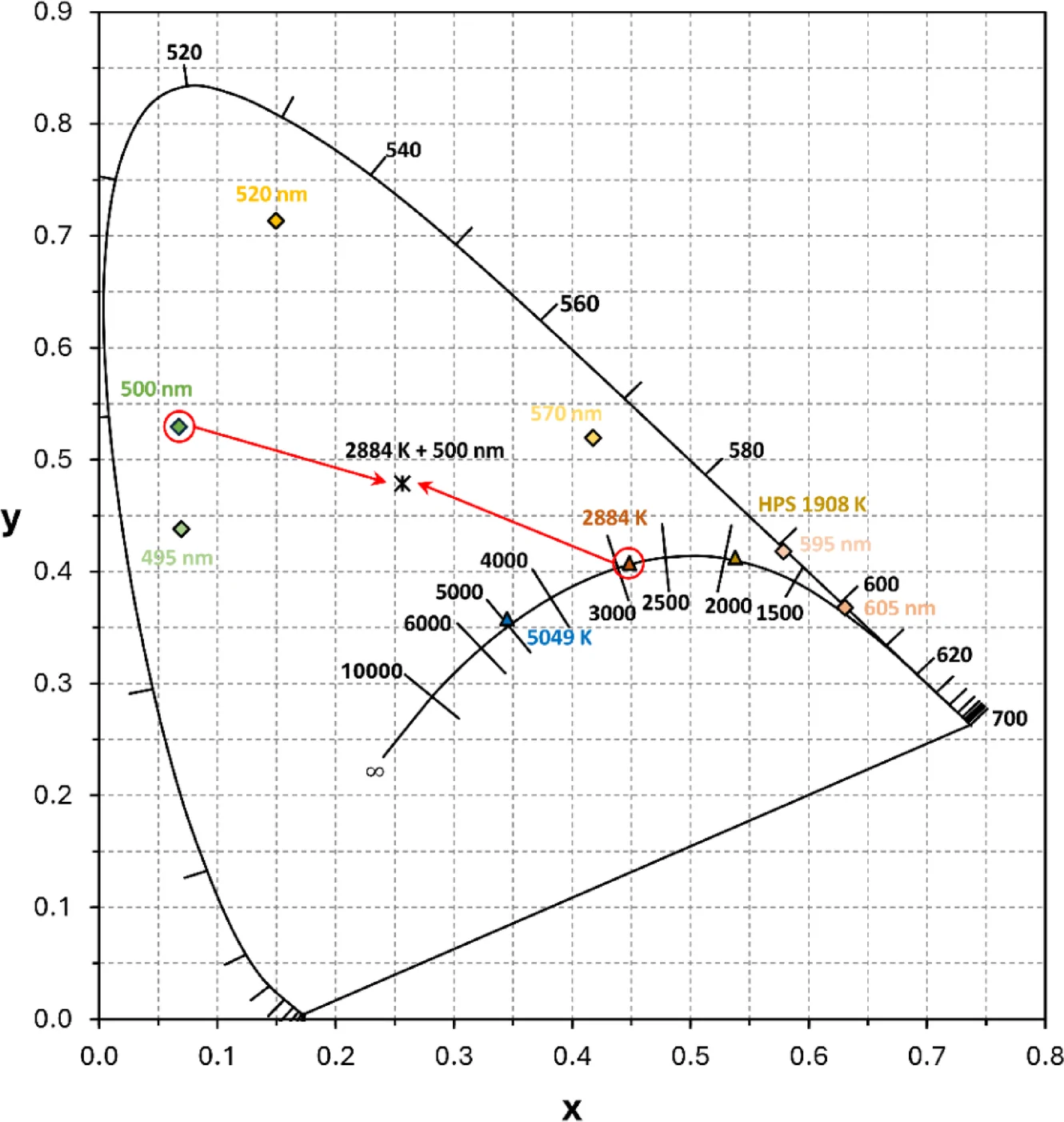
Radiation chromaticities of the tested quasi-monochromatic LED chips and other light sources used as reference or comparison (LED 2884 K, LED 5049 K, HPS 1908 K, mix 2884 K + 500 nm).

Figure 10 shows the chromaticity positions of the tested LEDs on the x, y chromaticity plane relative to the blackbody curve. The chromaticity points of the quasi-monochromatic LEDs lie in areas of high color saturation, indicating that they are located far from the blackbody locus. In the area where the CCT can be determined, the chromaticity points of the 595 nm and 605 nm LEDs are located. However, their mesopic luminous efficacy significantly decreases as the adaptation luminance decreases.

An interesting case is the mesopic luminous efficacy characteristic of the LED labeled “500 nm.” Its S/P ratio is 5.51 (Table 1). With a photopic luminous efficacy comparable to that of the 520 nm LED, the mesopic luminous efficacy of the 500 nm LED increases very rapidly, reaching 200 lm/W at an adaptation luminance level of 0.05 cd/m² and exceeding 300 lm/W at 0.005 cd/m². However, the color of this LED, described as “turquoise” under photopic vision conditions, may not be perceived particularly pleasant to the eye.

**Fig. 11:**
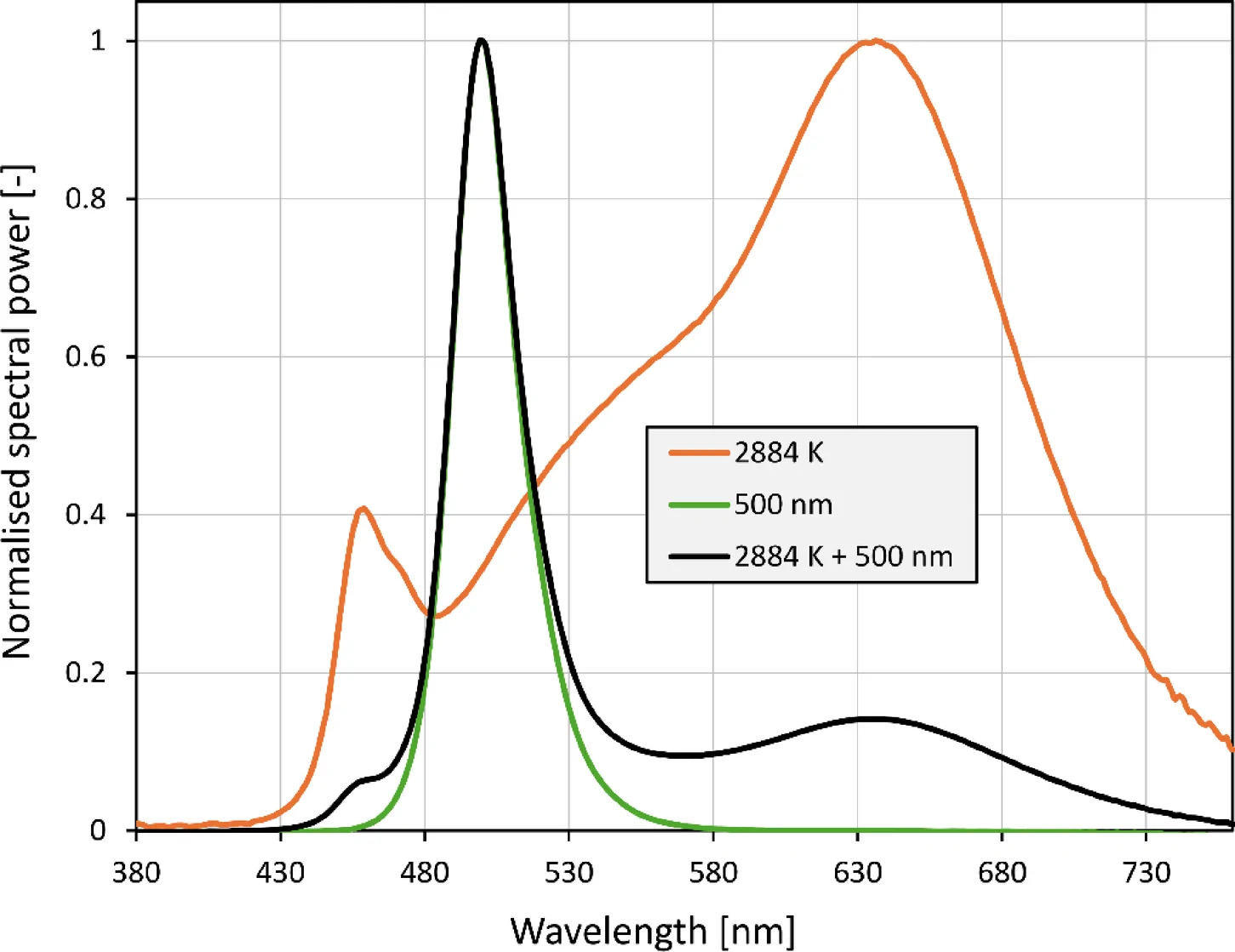
The spectral power distributions of the 500 nm and 2884 K diodes and the spectral distribution of the radiation created as a result of their mixing in a 1:1 ratio of electrical power.

**Fig. 12:**
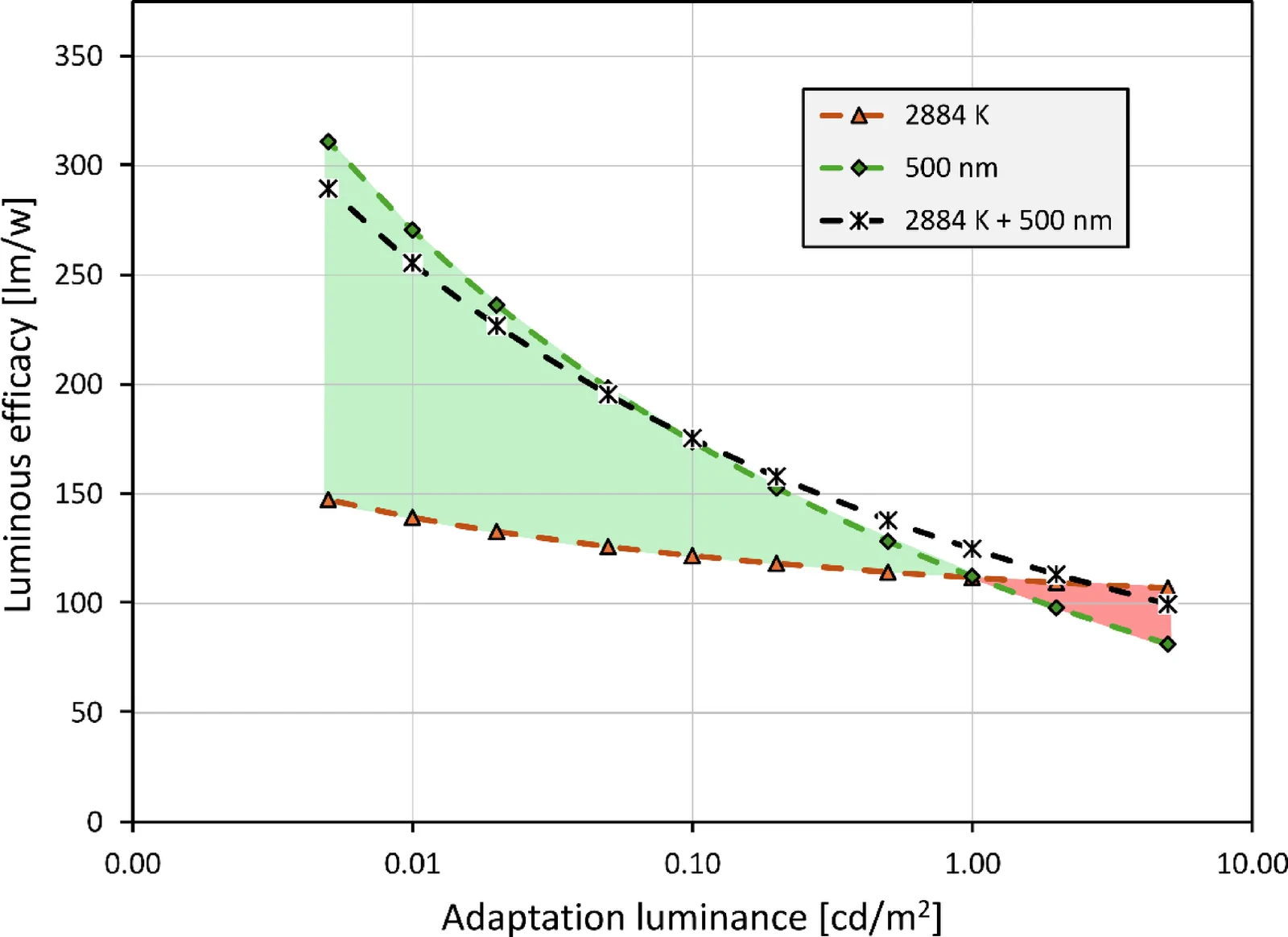
The characteristics of changes in luminous efficacy as a function of adaptation luminance for 500 nm and 2884 K diodes and the application emitting the spectral distribution of radiation created as a result of their mixing in a 1:1 ratio of electric power.

Therefore, it was decided to investigate the behavior of radiation obtained by mixing the light of this LED with that of a warm-white LED with a correlated color temperature of 2884 K in such proportions that half of the supplied power is used to generate white light and the other half to develop the 500 nm LED light. Furthermore, it should be emphasized that the Color Rendering Index (CRI) of this spectral power distribution is 42, which allows it to be successfully applied in outdoor areas in accordance with current lighting standards^35,37^. The SPD of the light emitted by this source combination is shown in Fig. 11. In contrast, the characteristic of mesopic luminous efficacy as adaptation luminance decreases is shown in Fig. 12. The chromaticity shift of the resulting light is indicated in Fig. 10.

The S/P ratio obtained by mixing the light of the 2884 K warm-white LED and the 500 nm LED is 3.77. The mesopic luminous efficacy of this radiation at an adaptation luminance of 0.05 cd/m² corresponds to 98.6% of the efficacy of the 500 nm LED. At the same time, the mesopic luminous efficacy of this mixture exceeds that of the 2884 K warm-white LED at adaptation luminance levels below 2 cd/m².

The light still exhibits a noticeable greenish tint, which may continue to be perceived as unpleasant for humans. This is because the chromaticity of the spectral mixture of 2884 K and 500 nm light remains far from the Planckian locus (Fig. 10), indicating a relatively high saturation of green. However, the literature reported that greenish light is much more suitable for birds^24^. Moreover, it should be noted that a high color saturation has not been an obstacle in the use of HPS lamps, which have effectively operated in road lighting installations and are even considered environmentally friendly in terms of light pollution compared to LED lighting^62^. In contrast, the 595 nm and 600 nm LEDs cannot be considered for improving the energy efficiency of outdoor lighting using quasi-monochromatic LEDs due to their low performance at approximately 0.1 cd/m² adaptation luminance.

### Energy efficiency assessment of quasi-monochromatic LED road lighting

To accurately assess the energy efficiency of quasi-monochromatic street lighting, the normalized power density (NPD) levels of road lighting implemented with the tested LEDs were calculated using typical formulas^63,64^. This was performed for hypothetical lighting scenarios as a function of road surface luminance levels ranging from 0.3 cd/m² to 2 cd/m². The increment of road surface luminance levels followed the EN 13,201 standard^32^: 0.3–0.5–0.75–1 – 1.5–2 cd/m².

**Fig. 13:**
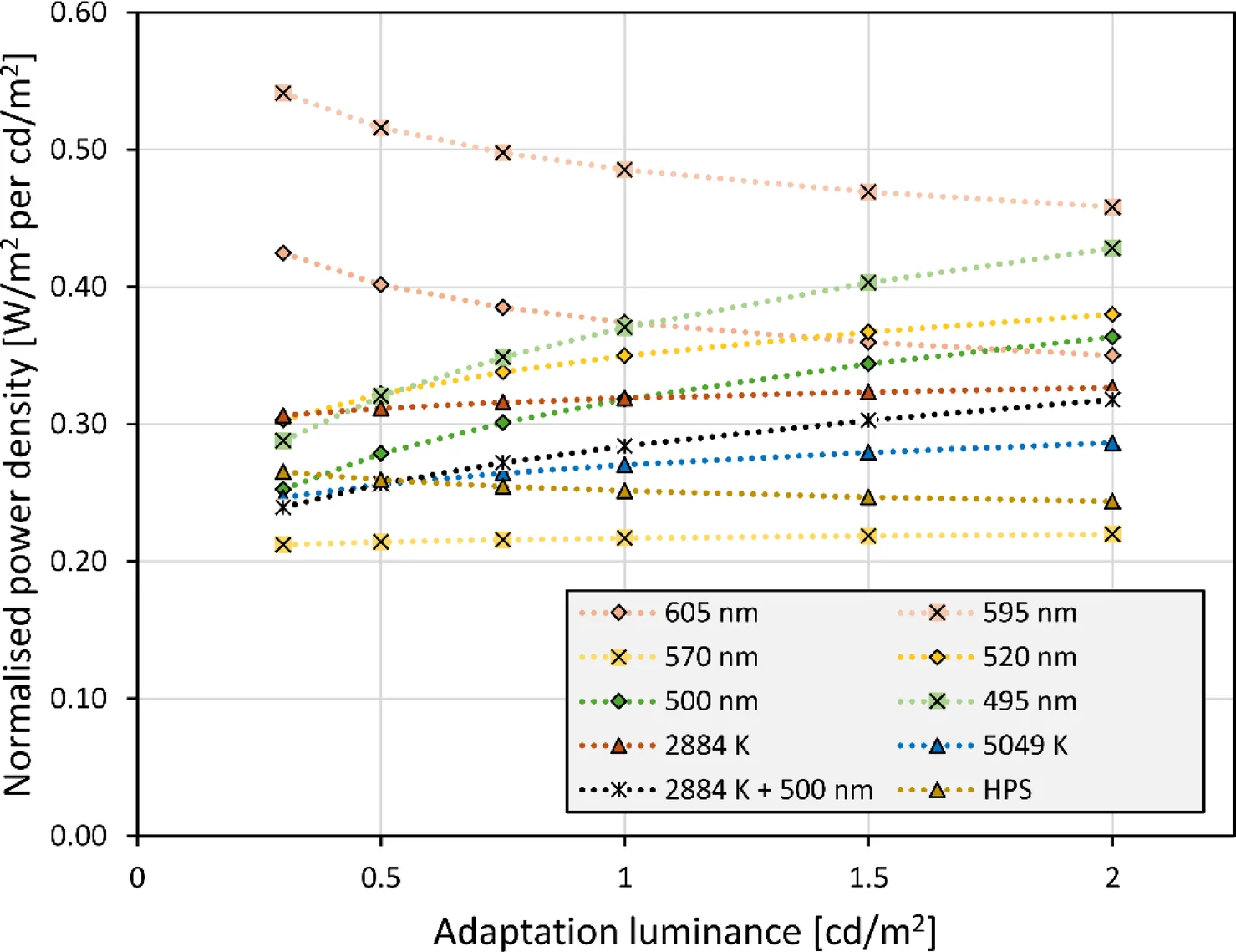
Normalized power density of road lighting as a function of road surface luminance for the tested samples.

The NPD levels were determined separately for each LED chip and other analyzed spectrums, assuming following parameters:

- road surface luminance coefficient q = 0.07,
- maintenance factor MF = 0.8,
- and a lighting utilization factor UF = 0.5.

The analysis also included lighting achieved by mixing the radiation of the 2884 K white LED with the 500 nm LED. The results of these calculations are presented in Fig. 13, which shows the dependence of NPD on adaptation luminance for each case analyzed.

The highest NPD levels, ranging from 0.46 W/m² per 1 cd/m² to 0.54 W/m² per 1 cd/m², were obtained for the LED chip with peak wavelength at 595 nm. The lowest NPD levels, from 0.21 W/m² per 1 cd/m² to 0.22 W/m² per 1 cd/m², were obtained for LED 570 nm. For lighting with the 595 nm and 605 nm LEDs, NPD values increase as the road surface luminance decreases. These increases do not exceed 10% for a one-step decrease in luminance level. The total increases amount to 18.1% and 21.3% when the luminance decreases from 2 cd/m² to 0.3 cd/m² for the 595 nm and 605 nm LEDs, respectively. For the remaining LEDs, NPD decreases with decreasing road surface luminance. These decreases do not exceed 10% when luminance decreases from 2 cd/m² to 0.3 cd/m² for the 570 nm LED and the 2884 K LED. The largest decreases in NPD for the same luminance change were observed for the 500 nm and 495 nm LEDs, amounting to 30.5% and 32.7%, respectively.

**Fig. 14:**
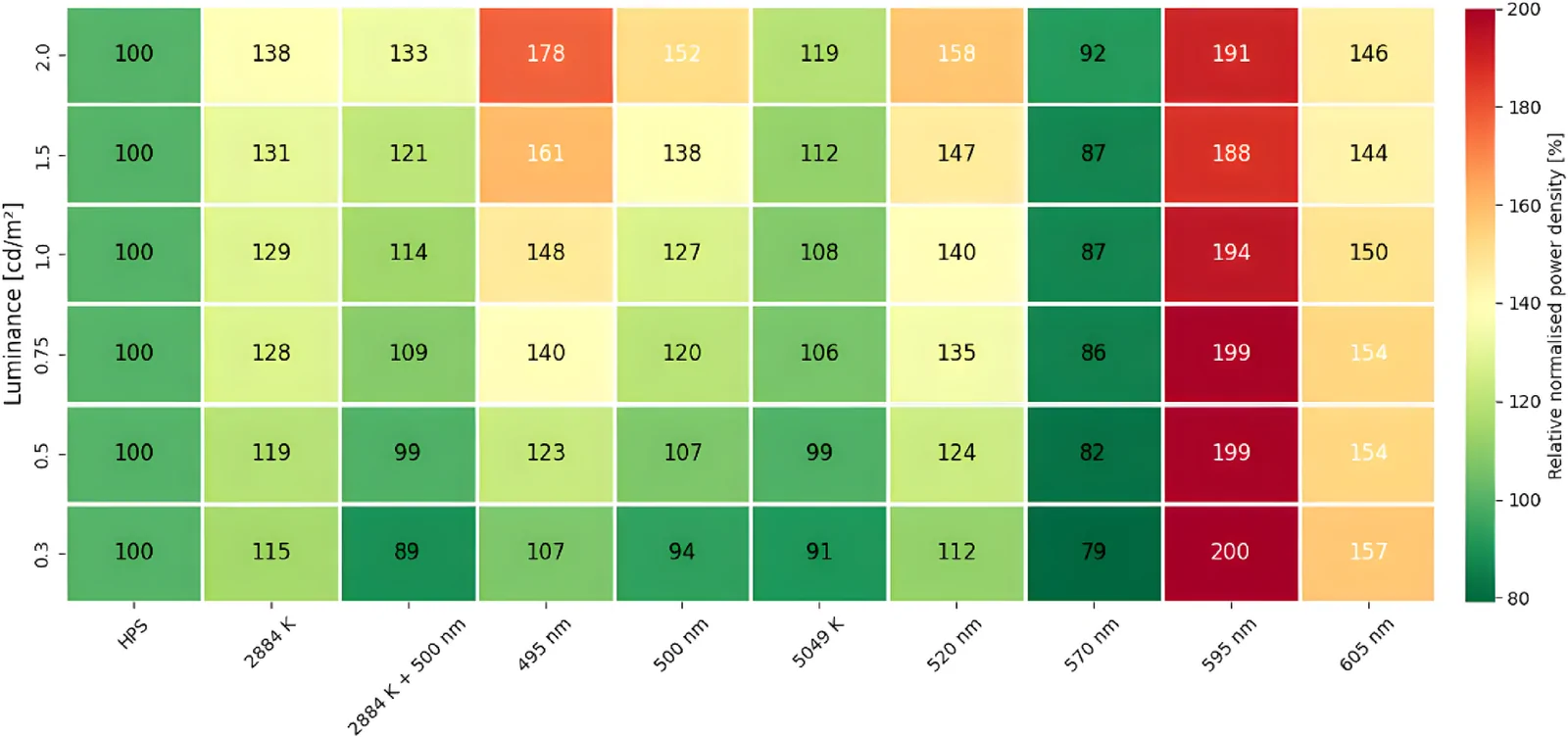
Relative normalized power density values at different lighting levels for selected samples; The HPS lamp is presented first from the left and serves as the reference (value 100). Values below 100 indicate energy savings, while values above 100 indicate energy waste.

The chart presented in Fig. 14 shows the relative NPD levels of road lighting for the individual LED chip, categorized by luminance levels ranging from 0.3 cd/m² to 2 cd/m². The reference value, set at 100% for each luminance level, is the NPD of the HPS lamp. At a luminance level of 0.3 cd/m², five LEDs are less efficient than the HPS lamp: 2884 K, 5049 K, 495 nm, 520 nm, 595 nm, and 600 nm. LEDs with wavelengths of 2884 K, 500 nm, and 570 nm can be more efficient by up to 20%.

For a luminance level of 0.5 cd/m², only the 570 nm LED is more efficient, by up to 18%. LEDs at 2884 K and 5049 K exhibit the same energy efficiency as the HPS lamp reference. At higher luminance levels, almost all tested LEDs show higher NPD levels compared to the HPS lamp. The exception is the 570 nm LED, which may be worth considering in combination with other white LEDs emitting low CCT to achieve further improvement in energy efficiency potential. The issue of mixing the spectral distributions of quasi-monochromatic LEDs with the spectra of white LEDs to determine an optimal, balanced lighting solution represents the main further research direction currently pursued by the authors.

**Fig. 15:**
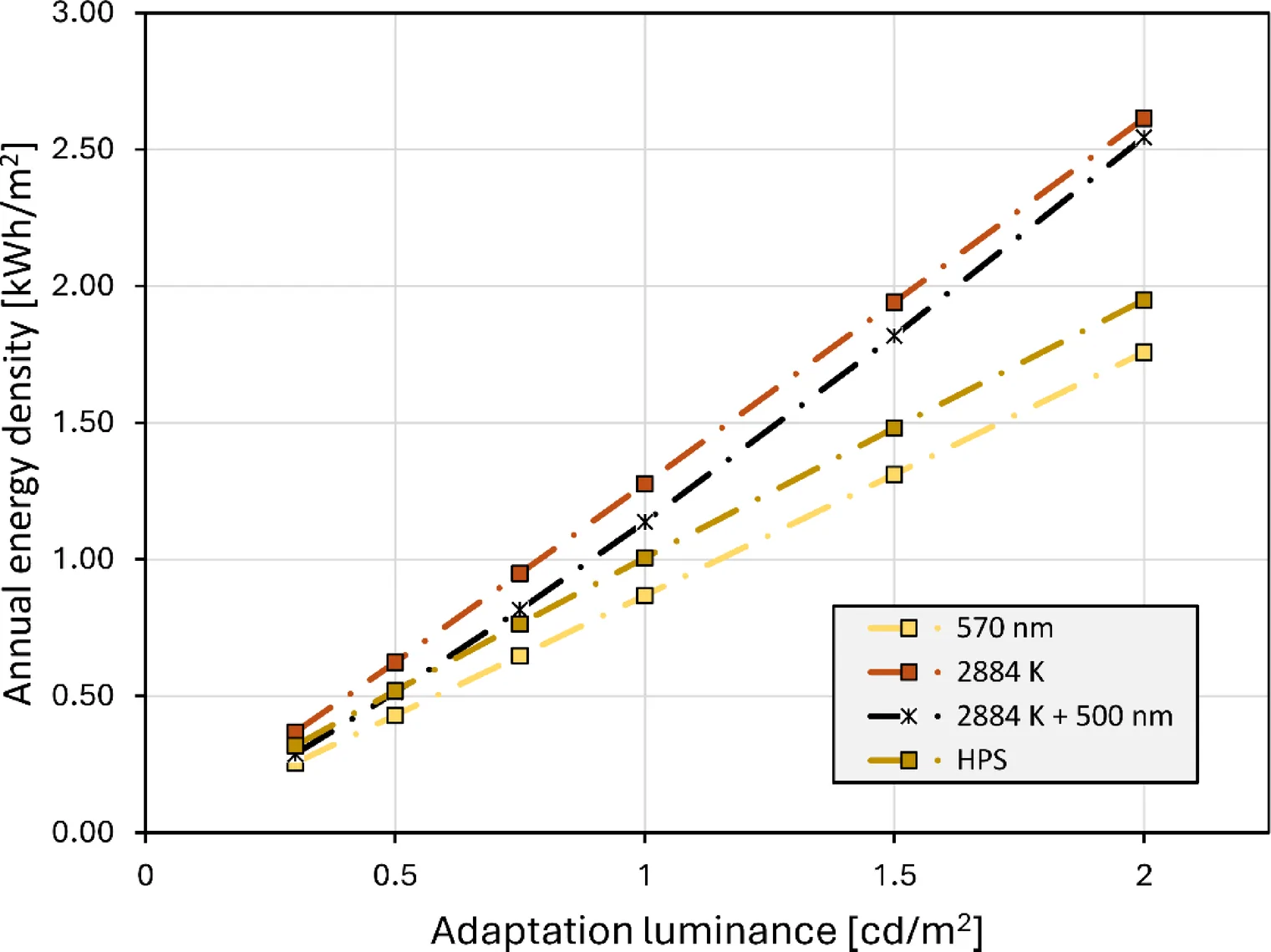
Annual energy density of road lighting as a function of road surface luminance, for LED 2884 K, LED 570 nm, LED 2884 K + LED 500 nm and HPS.

Additionally, to investigate the dynamics of energy efficiency, the annual energy density for road lighting W_D_ was also determined. It was done under the assumptions specified at the beginning of subsection 3.3, as well as for an annual operation time of 4000 h. The resulting W_D_ levels for luminance levels from 0.3 cd/m² to 2 cd/m², for lighting using the 570 nm LED, 2884 K LED, the 2884 K + 500 nm mixture, and the HPS lamp, are presented in Fig. 15.

The use of 570 nm LEDs and the 2884 K + 500 nm LED combination for road lighting, under mesopic vision conditions, results in energy savings compared to the 2884 K LED. Using the 2884 K + 500 nm LED combination provides savings ranging from 3% at a luminance level of 2 cd/m² to 22% at 0.3 cd/m². Using the 570 nm LED achieves savings from 31% at 0.3 cd/m² to 33% at 2 cd/m². The 2884 K + 500 nm LED combination is less advantageous than the 570 nm LED when compared to HPS lamps.

### Limitations and implications

It is important to highlight key research limitations and potential implementation issues in real-world conditions related to the proposed solution. It should be emphasized that a road lighting luminaire based on the selected type of quasi-monochromatic LEDs does not differ, in terms of operating principles and technical challenges, from a luminaire utilizing white LEDs. Consequently, appropriately selected constant-current drivers, optics (lenses), and control systems (dimming) as well as other accessories such as timers, motion sensors, or heat dissipation systems should be applied. It ensures that the solution requires no additional investment and is no more costly than conventional luminaires using white LEDs. Naturally, the implementation of an outdoor lighting luminaire utilizing quasi-monochromatic LEDs involves a meticulous research and development process to ensure that the lighting equipment meets the adopted standards and requirements. It also does not deviate from the requirements for white LED equipment and should be regarded as an advantage.

Moreover, other limitations of this study are limited number of LEDs tested and the lack of experimental studies using equipment implementing these LEDs. In real-world deployments of such systems, attention should be paid to visual contrast, especially at high color saturations different from those of HPS lamps, as well as the potential impact of the applied radiation on local flora and fauna. In road lighting, there are areas where color contrast is crucial in pedestrian crossing lighting, where the crossing area can be highlighted through color change^37^, or in fall prevention markings, which involve distinguishing steps and curbs using specialized yellow reflective tapes and paints^65^. However, in many situations, color contrast plays a secondary role to technical solutions based on specific shapes for traffic signs (circle, rectangle, triangle, diamond, etc.) or a fixed order of appearance, such as the red stop light always being at the top of a traffic signal. It ensures that individuals with color perception deficiencies (including color blindness) can participate safely and successfully in road traffic without posing a risk to others.

The issues described are significant and warrant further analysis. However, this requires conducting survey studies involving human participants, which is inherently time-consuming and challenging to execute. Nevertheless, such studies are planned for the future, as the present work describes the first stage of research and is the first in a series of articles dedicated to the comprehensive use of quasi-monochromatic LEDs in outdoor areas.

The results of this study are unique and clearly indicate that the proposed solution warrants further development and implementation in real-world conditions. Implementing road lighting solutions with quasi-monochromatic LEDs can yield substantial energy savings. The declared research aim has been achieved, and the proposed research hypothesis has been fully confirmed. Nevertheless, future research will be continued in the following areas:

- the use of quasi-monochromatic light for road lighting in combination (mixing) with white light,
- the impact of chromaticity on detail recognition,
- the impact of chromaticity on color sign recognition,
- the impact of colored lighting on environmental light pollution,
- the impact of colored lighting on the well-being of road users.

## Conclusions

This study contains key results from the initial stage of research into the potential use of quasi-monochromatic light to reduce energy consumption in outdoor lighting. It describes quantitative analyses of the energy demand required to generate a level of perceived brightness consistent with expectations under conditions in which the human eye activates all light receptors to perceive details in the surroundings. The development of quasi-monochromatic LEDs is not as rapid as that of white LEDs, and in the vast majority of cases, the luminous efficacies of quasi-monochromatic LEDs cannot compete with white LEDs. Although white LEDs emit radiation across a wide range of wavelengths, which produces a significantly weaker perception of brightness than the radiation from many monochromatic LEDs, white LEDs achieve very high efficiency in converting electrical energy into light.

While the following observations bring the research closer to its objective, a definitive prescription for the specific implementation of quasi-monochromatic light remains to be established. Nevertheless, quasi-monochromatic LED light demonstrates significant potential, particularly for applications involving low or very low levels of adaptation luminance. Excellent examples are the tested quasi-monochromatic LEDs with emission peaks at 500 nm and 570 nm. The 500 nm LED can be competitive when combined with white LEDs. In comparison, the 570 nm LED can be competitive thanks to its light-generation mechanism, which is the same as that used in white LEDs. Moreover, supplementing typical white LEDs with quasi-monochromatic light (mix 2884 K + 500 nm) provides color rendition that is not worse than that of HPS lamps, which were commonly used until recently.

Quasi-monochromatic LED technology has high development potential. Achieving luminous efficacies comparable to or even exceeding white LEDs is entirely possible, but it requires further advancement in monochromatic LED manufacturing. This technology offers the possibility of significant energy savings in road lighting, especially for roads with the lowest required lighting levels located in dark or very dark surroundings. Energy savings in road lighting, with reference to high pressure lamps, using available monochromatic LEDs, under mesopic vision conditions, can reach approximately 20% level.

## Data Availability

Data underlying the results presented in this paper are not publicly available at this time but may be obtained from the authors upon reasonable request.

## List of symbols

*CCT*: Correlated color temperature [K]
*CRI*: Color rendering index [-]
*Duv*: Distance from the Black Body Locus
${E}_{v,p}$: Photopic illuminance [lx]
*FWHM*: Full width at half maximum (for spectrum)
*HPS*: High pressure sodium (lamp)
${K}_{p}$: Photopic radiation equivalent equal to 683 lm/W
${K}_{s}$: Scotopic radiation equivalent equal to 1699 lm/W
*LED*: Light emitting diode
${L}_{A}$: Adaptation luminance [cd/m^2^]
$${L}_{e}\left(\lambda \right)$$: Radiance [Wsr^−1^m^−2^]
${L}_{v,m}$: Mesopic luminance [cd/m^2^]
${L}_{v,p}$: Photopic luminance [cd/m^2^]
${L}_{v,s}$: Scotopic luminance [cd/m^2^]
*LID*: Light intensity distrubition (curve)
*MF*: Maintenance factor [-]
*m*: Adaptation coefficient [-]
${m}_{0}$: Initial adaptation coefficient from CIE191 report equal to 0.5
${m}_{n-1}$: Adaptation coefficient in step n-1
${m}_{n}$: Adaptation coefficient in step n
*NPD*: Normalized power density [W/m^2^ per 1 cd/m^2^]
*P*: Active power [W]
*q*: Luminance coefficient of road surface [sr^−1^]
*S/P*: Scotopic to photopic index [-]
*SPD*: Spectral power distribution (of radiation)
${\phi}_{e}\left(\lambda \right)$: Spectral power distribution of radiant flux [W/nm]
${\phi}_{p}$: Photopic luminous flux [lm]
${\phi}_{s}$: Scotopic luminous flux [lm]
*UF*: Utilisation factor [-]
$V\left(\lambda \right)$: Photopic luminous efficiency of the human eye [-]
${V}^{^{\prime}}\left(\lambda \right)$: Scotopic luminous efficiency of the human eye [-]
${W}_{D}$: Annual energy density [kWh/m^2^]
${V}_{mes}\left(\lambda \right)$: Mesopic luminous efficiency of the human eye for the given adaptive luminance [-]
*η*: Photopic luminous efficacy [lm/W]
${\eta}_{mes}$: Mesopic luminous efficacy [lm/W]
*λ*: Wavelength [nm]
*x,y*: Chromaticity coordinates [-]
